# Characterization of Novel Mycobacteriophages of Subcluster A3: Evolutionary Plasticity of the Lysogeny Module and Lytic Potential

**DOI:** 10.3390/ijms27198476

**Published:** 2026-09-23

**Authors:** Marina Zaychikova, Maja Malakhova, Sofya Kuznetsova, Dmitry Bespiatykh, Sergei Kiselev, Ksenia Klimina, Aleksandra Strokach, Arina German, Anastasiia Lebedeva, Mikhail Fursov, Anna Vnukova, Dmitry Bagrov, Nadezda Trofimova, Margarita Shleeva, Egor Shitikov

**Affiliations:** 1 Lopukhin Federal Research and Clinical Center of Physical-Chemical Medicine of the Federal Medical Biological Agency, Moscow 119435, Russia; majamalakhova@rcpcm.ru (M.M.); sofya.kuznetsova.2001@list.ru (S.K.); kiselev.si@phystech.edu (S.K.); alexandra.vlasova.2017@yandex.ru (A.S.); fursov@obolensk.org (M.F.); bagrov@mail.bio.msu.ru (D.B.); nadya188@bk.ru (N.T.); margoshleeva@gmail.com (M.S.); eshitikov@rcpcm.ru (E.S.); 2 Moscow Center for Advanced Studies, Moscow 123592, Russia; 3 Faculty of Biology, Lomonosov Moscow State University, Moscow 119991, Russia; vnukovaaa@my.msu.ru; 4 Federal Research Centre ‘Fundamentals of Biotechnology’ of the Russian Academy of Sciences, Moscow 119071, Russia; gerarinaman@yandex.ru; 5 State Research Center for Applied Microbiology and Biotechnology, Obolensk 142279, Russia; ay_lebedeva@mail.ru

**Keywords:** mycobacteriophage, subcluster A3, one-step growth curve, lysogeny

## Abstract

The increasing prevalence of drug-resistant *Mycobacterium* *tuberculosis* (*Mtb*) and nontuberculous mycobacteria (NTM) underscores the need for novel strategies. Mycobacteriophages are attracting growing interest for therapeutic and diagnostic applications. Here, we describe four new subcluster A3 phages (Cat, Goose1D, Goose1X, and Shep) and investigate their genomic diversity and lytic activity. The phages share >98% nucleotide identity with known A3 members but carry distinct deletions within the lysogeny module: Cat and Goose1X have different repressor-loss variants, and Goose1D lacks the integrase gene, whereas Shep retains an intact module. All phages lysed *M. tuberculosis* H37Rv, with efficiencies of plating (EOPs) of 10^−2^–10^−1^, but showed limited activity against NTM. Cat was the most effective against drug-resistant *Mtb* isolates (EOPs of 10^−1^–1), remained stable across a broad pH and temperature range, but had a low burst size (8 PFU (plaque-forming units/cell). In lysogeny assays, 6% of Cat-exposed *M. smegmatis* colonies exhibited a lysogen-like phenotype, but no phage DNA was detected, suggesting spontaneous resistance. Sequencing of one strain revealed an H535Q substitution in the pyruvate carboxylase MSMEG_2412. These findings highlight lysogeny module plasticity in closely related phages and position Cat, with its naturally occurring repressor deletion and potent lytic activity, as a promising candidate against drug-resistant tuberculosis.

## 1. Introduction

The global spread of *Mycobacterium tuberculosis* (*Mtb*) and pathogenic nontuberculous mycobacteria (NTM) represents a major threat to public health [1]. Despite the availability of numerous antitubercular drugs, treatment remains prolonged, complex, and toxic, while the increasing prevalence of drug-resistant strains demands fundamentally new therapeutic strategies [2,3]. In this context, phage therapy, which uses bacteriophages as natural bacterial antagonists, has attracted considerable interest.

Mycobacteriophages have been studied since the mid-twentieth century. More than 11,000 mycobacteriophages have now been identified, of which at least 2500 have been sequenced and annotated. These phages are classified into 36 clusters [4,5]. Mycobacteriophages lacking sufficient similarity to any established cluster are classified as singletons, of which six are currently recognized. Despite the large number of known phages, their geographical distribution is highly uneven, with the vast majority isolated in North America [6,7].

Mycobacteriophage genomes range from 42 to 164 kb in length, with a mean size of approximately 70 kb. Most have siphovirus morphology, comprising a long, non-contractile tail and an icosahedral capsid, although phages in clusters O, I, and Y have elongated capsids. Clusters C and AA form a distinct group: all known members of these clusters exhibit myovirus morphology [5]. Mycobacteriophages can demonstrate either lysogenic or strictly lytic life cycles. Phages within a given cluster generally share the same life-cycle strategy, with lysogeny predominating in most clusters.

Although most mycobacteriophages were isolated using the laboratory strain *M. smegmatis* mc(2)155, some are also active against pathogenic mycobacteria. Successful clinical use of phages has been reported for NTM infections, particularly in individuals with cystic fibrosis or immunodeficiency, for whom conventional antibiotic therapy is often ineffective [8]. In particular, phages have shown activity against *M. abscessus*, *M. chelonae*, and *M. avium*.

In contrast to NTM infections, no clinical cases of phage therapy for tuberculosis have yet been reported. Nevertheless, preclinical studies provide compelling evidence for the potential of this approach. A cocktail of five lytic phages showed potent in vitro bactericidal activity against a broad panel of clinical *Mtb* isolates [9]. Phage treatment has also demonstrated efficacy against tuberculosis in C57BL/6 mice following either aerosol or intravenous administration [10].

Most mycobacteriophages with therapeutic potential, including those capable of infecting *Mtb*, belong to a limited number of clusters, predominantly G, K, AB, and A [9,11]. Cluster A is the largest and consists mainly of temperate phages, comprising 903 known members distributed among 22 subclusters (A1–A22). The majority of these belong to subclusters A1 (241 isolates), A4 (146), A3 (134), and A2 (123), whereas subclusters A17–A22 each contain only one or two phages (https://phagesdb.org/clusters/A/; accessed 15 July 2026). Lytic activity against *Mtb* is not a universal trait within cluster A but has been demonstrated at least for subclusters A2 and A3 and for a small number of A1 representatives [11].

Phages L5 and D29, both assigned to subcluster A2, are among the best-characterized cluster A phages. The temperate phage L5 was isolated in Japan in 1960 and was the first mycobacteriophage to be sequenced [12], establishing a foundation for understanding the genomic organization of cluster A. D29 attracted particular interest because it lost the capacity for lysogeny following deletion of its repressor gene [13]. It nevertheless retained a broad host range and efficiently infects both rapidly and slowly growing mycobacteria, including *Mtb* [14]. These properties have led to the widespread use of D29. Luciferase reporter phages derived from D29 have been developed to diagnose drug resistance [15,16], and the phage has shown therapeutic efficacy both in mouse models of tuberculosis [9] and in clinical treatment of NTM infections [8].

Although the inability to form lysogens has been experimentally confirmed only for D29, approximately 10% of subcluster A2 phages produce clear plaques characteristic of lytic phages [5]. This suggests that loss of the repressor may not be a rare evolutionary event within cluster A.

Here, we isolated four new subcluster A3 mycobacteriophages, Cat, Goose1D, Goose1X, and Shep, from environmental samples collected in Russia. Using comparative genomics, we examined the organization of their lysogeny modules and identified distinct deletion events affecting the integrase and repressor genes. Host-range analysis was used to assess their lytic activity against *Mtb* and NTM, leading us to select Cat as the most promising candidate for further biological characterization, including analysis of its life-cycle parameters and stability. We also investigated the origin of phage-resistant mutants recovered during lysogeny-frequency assays. These findings expand our understanding of genomic plasticity within subcluster A3 and identify Cat, with its naturally occurring deletions in the lysogeny module, as a promising candidate for further investigation, including potential biomedical applications.

## 2. Results

### 2.1. General Characteristics and Phylogenetic Placement of the Phages

Four mycobacteriophages were isolated, each producing plaques 3–5 mm in diameter with turbid margins on *M. smegmatis* mc(2)155 (Figure 1). Transmission electron microscopy showed that the phages had similar siphovirus morphology, with icosahedral capsids measuring 66 ± 3 nm in diameter and long, non-contractile tails measuring approximately 160 ± 8 nm and terminating in a prominent baseplate.

Whole-genome sequencing showed that the phages had similar genome sizes (45.6–48.4 kb), G + C contents (~63.6–63.9%), and numbers of predicted open reading frames (ORFs; 71–77) (Table 1). Two *tRNA* genes (*tRNA-Asn* and *tRNA-Trp*) were identified in each of the three phages, whereas Cat had only one (*tRNA-Trp*).

Phylogenetic analysis assigned all four phages to subcluster A3 (genus *Veracruzvirus* under the current ICTV taxonomy [MSL41.v1; Ref. [17]) (Figure 2). Shep, Goose1X, and Goose1D clustered with Heathen (MN062706), HelDan (NC_042328), Fred313 (MF373840.1), and Scout (MW712718.1). These relationships were supported by high nucleotide sequence identity: >99.9% among Shep, Goose1X, and Goose1D and ~98% between these phages and the related reference genomes. Cat was most closely related to BabyRay (NC_054772; ~99% identity) and showed lower identity to the other three phages under study (91.8–93.8%).

### 2.2. Functional Annotation of the Phage Genomes

Genome annotation revealed an organization typical of siphoviruses [18]. Genes encoding virion structural and assembly proteins were located on the left arm and transcribed in the forward direction, whereas genes involved in nucleic acid metabolism and regulation of lysogeny were located on the right arm and transcribed in the reverse direction. The lysis cassette contained two components, lysins A and B. No holin gene was identified, consistent with other phages in this subcluster. The remaining predicted genes encoded proteins involved in host metabolism and integration, as well as numerous hypothetical proteins.

A notable feature shared by these phages was the presence of large genomic deletions. Relative to Heathen (Figure 3A), Shep contained a 2534-bp deletion on the right arm that removed 11 genes adjacent to the transcriptional repressor (genes 76–86; positions 45,221–47,754), all encoding hypothetical proteins. Goose1X carried a 4025-bp deletion encompassing nearly the entire region downstream of the repressor on the right arm (genes 73–87; positions 44,278–48,302), except for the two most distal genes. Its repressor was truncated and retained only an 89-amino-acid C-terminal domain. In Goose1D, a 3043-bp deletion in the central genomic region removed seven genes (genes 32–38; positions 24,924–27,966), encoding a lipoprotein, tyrosine integrase, membrane protein, helix–turn–helix domain-containing protein, dCMP deaminase, and two hypothetical proteins.

The most notable difference between Cat and BabyRay was a 2056-bp deletion on the right arm of the Cat genome (genes 74–80; positions 44,082–46,137) that disrupted the transcriptional repressor, resembling the deletion described in the lytic phage D29 (Figure 3B). In addition to the repressor, this deletion removed six downstream genes encoding hypothetical proteins.

### 2.3. Phage Host Range

All four phages demonstrated the ability to efficiently lyse *M. tuberculosis* H37Rv (Figure 4). They produced large zones of lysis on bacterial lawns: Cat and Goose1X formed completely clear zones, whereas those produced by Shep and Goose1D were turbid. Goose1X, Goose1D, and Shep each had an efficiency of plating (EOP) of no greater than 10^−2^, whereas Cat showed the highest EOP at 10^−1^. Cat was subsequently tested against five drug-resistant *Mtb* isolates representing distinct phylogenetic lineages and produced EOP values ranging from 10^−1^ to 1 (Figure S1).

The phages showed negligible or no activity against the tested NTM isolates (*M. abscessus*, *M. avium*, *M. fortuitum* and *M. kansasii*), with EOP values no greater than 10^−6^ (Figure 4).

### 2.4. Characterization of Phage Cat

To better characterize phage Cat, which showed the highest EOP on *Mtb*, we examined its life-cycle parameters and stability under various conditions. Adsorption analysis showed that >90% of phage particles bound to *M. smegmatis* mc(2)155 cells within the first 40 min after infection (Figure 5A). One-step growth analysis revealed a latent period of 60 min, followed by a 90-min rise period (Figure 5B). The growth curve reached a plateau at 150 min, and the burst size was eight plaque-forming units (PFU) per infected cell.

Phage Cat was also tested for stability across a range of pH values, temperatures, and UV exposure (Figure 5C). It retained infectivity from pH 4 to pH 11, whereas its activity was almost completely lost at pH 3 and pH 12. The phage remained active at temperatures ranging from −20 °C to 45 °C but was completely inactivated at 55 °C. Ultraviolet irradiation caused progressive phage inactivation. The titer declined most rapidly during the initial phase of exposure, after which the rate of inactivation decreased. By the end of the experiment, infectivity had declined by six orders of magnitude.

### 2.5. Lysogeny and Superinfection Immunity

To investigate the apparent lytic phenotype of phage Cat, its ability to form lysogens in *M. smegmatis* mc(2)155 was assessed. Colonies phenotypically resembling lysogens arose at frequencies of up to 6%. Further analysis of the recovered cells revealed broad resistance, extending not only to subcluster A3 phages but also to the unrelated subcluster K4 phage YPΔ47 (Figure 6). By comparison, Shep, which served as a control, produced lysogens at a frequency of 10.5%. Lysogens were resistant only to subcluster A3 phages.

To determine whether the mc(2)155(Cat) phenotype resulted from stable lysogeny or from phage resistance, we performed two independent PCR assays on individual colonies: (i) PCR using primers flanking the predicted phage integration site (MSMEG_4676, *tRNA*-*Gly*) and (ii) PCR with Cat-specific primers (Figure S2). For mc(2)155(Shep), the integration-site PCR produced an amplicon, confirming the presence of a prophage, whereas no amplification was observed for mc(2)155(Cat) in either assay. Whole-genome sequencing of one mc(2)155(Cat) clone and the parental *M. smegmatis* mc(2)155 strain further confirmed the absence of phage DNA and revealed a single nucleotide polymorphism in MSMEG_2412 (g2494665c), resulting in a histidine-to-glutamine substitution at codon 535 (H535Q).

## 3. Discussion

Here, we characterized four new subcluster A3 phages, representing the first members of this subcluster isolated in Eurasia. Previously, the majority of subcluster A3 phages had been isolated in North America, with only one isolate recovered in New Zealand (Actinobacteriophage Database, as of 15 July 2026). Despite this geographical distance, the phages described here show high genetic similarity to known North American isolates. This finding is consistent with the global distribution of closely related mycobacteriophages and the absence of strict geographical structuring among their genomes [19,20].

Beyond their global phylogeography, these phages are notable for their genomic organization. All four isolates carry large deletions, some of which affect the lysogeny module. Shep, Goose1D, and Goose1X are particularly informative in this regard. Although isolated from nearby samples and nearly identical at the genomic level (>99.9%), the phages carry distinct deletions. This pattern suggests that the deletion events arose recently and independently and, in the cases of Goose1D and Goose1X, is consistent with the frequent loss of lysogeny among cluster A phages [5,18].

To place these findings in a broader context, we analyzed 117 subcluster A3 genomes and identified three distinct repressor variants (Figure S3). Within-variant nucleotide identities ranged from 89.0% to 100%, whereas between-variant identities were substantially lower (65.0–86.2%) (Figure S3). The first variant was characteristic of the genus *Microwolfvirus*. By contrast, variants 2 and 3 showed high similarity to each other, reflecting their closer relationship within the *Veracruzvirus* phages. Notably, repressor deletions and potentially inactivating mutations were found in all three variants: complete repressor deletions in Misomonster, MK4, Cat, and Caviar; partial repressor loss in Zetzy, AgronaGT15, Kalb97, Phranny, Goose1X, and Rockstar; and single-nucleotide indels in the repressor genes of Grub, Wonder, Bxz2, BuzzBuzz, Louie6, JF4, JF2, Phantastic, MA5, Veracruz, and Noella. Of these, only Bxz2 has been experimentally confirmed to lose lysogen-forming capacity due to a frameshift [19]. The functional consequences of the other indels remain unresolved, and some may represent genome assembly artefacts. Nevertheless, the diversity of repressor-loss variants across all three repressor types highlights the pronounced evolutionary plasticity of the lysogeny module in subcluster A3.

Integrase deletion appears to be a substantially rarer evolutionary event. Among the 117 genomes analyzed, 89 encoded a tyrosine integrase, 27 a serine integrase, and in only one case (Goose1D), the integrase gene was completely deleted (Figure 2). Notably, phages with tyrosine integrases appear to integrate into the *tRNA*-*Gly* site (MSMEG_4676), as demonstrated here for Shep, which is also used by subcluster A2 phages [18]. By contrast, phages with serine integrases, such as Bxz2 (*Microwolfvirus*), use a different integration site (MSMEG_5156) consisting of only 4 bp, which was identified experimentally [21]. However, whether the same site is used by serine integrases of the genus *Veracruzvirus* remains to be determined, as the integrases of these two genera share less than 30% amino acid identity.

The prevalence of such alterations in the lysogeny module, together with the apparently high frequency of these events demonstrated here, makes representatives of the A3 subcluster attractive objects for potential therapeutic and diagnostic applications against mycobacterial infections. In addition to the previously demonstrated activity of cluster A phages against pathogenic mycobacteria, it is important to emphasize that the deletions identified here are naturally occurring, which may facilitate their regulatory approval. Despite these deletions, all four phages retained the capacity to infect *Mtb*, with EOP values of 10^−2^–10^−1^, indicating that the deleted genes are non-essential for productive infection under the conditions tested. Cat showed the greatest activity and efficiently lysed drug-resistant *Mtb* isolates. Comparison with published data places its efficiency within the range reported for highly active subcluster A3 phages, including Bxz2, Microwolf and Vix (EOP = 10^−1^–5.0). By contrast, A3 phages HelDan and JHC117 show extremely low efficiencies of infection (<10^−7^) [11]. We found no association between the efficiency of *Mtb* infection and phylogenetic position within subcluster A3.

Phage Cat remained active across broad temperature and pH ranges, as reported for many other mycobacteriophages [22,23]. These findings indicate sufficient physicochemical stability for potential practical applications, although storage conditions will require careful consideration, particularly with regard to prolonged ultraviolet exposure. Despite its high lytic activity and stability, Cat had a low burst size of only eight PFU per infected cell, substantially lower than the 10^4^ PFU/cell reported for D29 [24]. We found no published burst-size data for other subcluster A3 phages; it therefore remains unclear whether this low yield is characteristic of the subcluster or specific to Cat.

Another notable feature of the interaction between Cat and *M. smegmatis* was the emergence, at a frequency of 6%, of colonies phenotypically resembling lysogens. No phage DNA was detected in the individual colonies tested, indicating that they represented resistant mutants rather than true lysogens. Whole-genome sequencing of one clone identified a mutation in MSMEG_2412, which encodes pyruvate carboxylase and has not previously been associated with phage resistance. Given that this enzyme contributes to central metabolism and may influence cellular energy status [25], the mutation could potentially affect host processes required for successful infection by cluster A3 phages. Notably, adsorption of Cat phage to the resistant mutant was comparable to that of the parental strain, pointing to an intracellular mechanism of resistance rather than a defect in phage attachment (Figure S4). However, the precise mechanism remains to be elucidated.

To distinguish a stable genetic change from transient bacterial gene overexpression, such as that described for *mpr* during D29 infection [26], the resistant strain was passaged ten times in the absence of phage. Resistance was retained, supporting a stable genetic basis rather than a transient physiological response. However, because only one resistant clone was subjected to whole-genome sequencing, the possibility that other colonies acquired resistance through different mechanisms cannot be excluded.

## 4. Materials and Methods

### 4.1. Bacterial Strains and Culture Conditions

*M. smegmatis* mc(2)155 and *M. tuberculosis* H37Rv were obtained from the collection of the A.N. Bach Institute of Biochemistry, Russian Academy of Sciences. Clinical isolates of *M. abscessus*, *M. fortuitum*, *M. avium*, and *M. kansasii* were provided by the National Medical Research Center of Phthisiopulmonology and Infectious Diseases, Ministry of Health of the Russian Federation. A panel of clinical *Mtb* isolates was obtained from the State Collection of Pathogenic Microorganisms (SCPM–Obolensk).

Mycobacterial strains were cultivated as described previously [27] using Middlebrook 7H9 broth (HiMedia, Thane, India) supplemented with 0.05% Tween-80 (Sigma-Aldrich, St. Louis, MO, USA) and Middlebrook 7H11 agar (HiMedia, Thane, India), both enriched with 10% Middlebrook OADC Supplement (HiMedia, Thane, India). Tween 80 was omitted when culturing *M. smegmatis* mc(2)155. Middlebrook 7H9 soft agar containing 0.7% agar was used for phage experiments. CaCl_2_ was added to all media to a final concentration of 2 mM for *M. smegmatis* mc(2)155 and 1 mM for all other mycobacteria. Cultures were incubated at 37 °C in a humidified atmosphere containing 5% CO_2_; liquid cultures were agitated at 120 rpm. Experiments involving pathogenic mycobacteria were conducted under Biosafety Level 3 containment in accordance with national biosafety guidelines.

### 4.2. Isolation of Mycobacteriophages

Phages Cat, Goose1D, Goose1X, and Shep were isolated from environmental samples collected in the Ramensky district of the Moscow region using an enrichment-culture method described previously [27]. Cat was recovered from soil collected at a site inhabited by stray cats. Goose1D and Goose1X were isolated from litter collected at a poultry enclosure housing waterfowl, whereas Shep was isolated from sheepfold bedding.

Each sample (10 g) was incubated overnight in 10 mL MP buffer (50 mM Tris–HCl, 150 mM NaCl, 10 mM MgCl_2_, 2 mM CaCl_2_ and 0.1 mg/mL ampicillin, pH 7.5) supplemented with a mid-logarithmic-phase culture of *M. smegmatis* mc(2)155. Samples were centrifuged at 5000× *g* for 20 min, and the supernatants were filtered through 0.22-µm PES membrane filters (Millipore, Burlington, MA, USA). Filtrate was mixed 1:1 with twofold-concentrated tryptic soy broth (HiMedia, Thane, India), inoculated with *M. smegmatis* mc(2)155 and incubated with continuous agitation for 48 h. Phages were purified by three successive rounds of single-plaque isolation using the double-agar overlay method [28].

### 4.3. Transmission Electron Microscopy

Phage particles were concentrated by ultracentrifugation in an SW 55 Ti rotor at 23,000 rpm for 2 h at 20 °C using an Optima XPN-90 ultracentrifuge (Beckman, Brea, CA, USA). Pellets were resuspended in SM buffer (50 mM Tris–HCl, 100 mM NaCl, 8 mM MgSO_4_ and 0.01% gelatin, pH 7.5). Samples were applied to carbon-coated grids (Ted Pella, Redding, CA, USA) that had been treated using a K100X glow-discharge system (Quorum Technologies Ltd., Laughton, UK), followed by negative staining with 1% aqueous uranyl acetate. Samples were visualized using a JEM-1400 transmission electron microscope (JEOL, Tokyo, Japan) operated at an accelerating voltage of 120 kV.

### 4.4. Phage Adsorption Assay

Phage adsorption to the parental *M. smegmatis* mc(2)155 strain and the phage-resistant mutant mc(2)155(Cat) was assessed as described previously [29]. Mid-logarithmic-phase cells were mixed with phage at a multiplicity of infection (MOI) of 0.01 in Middlebrook 7H9 broth to a final volume of 5 mL. During the 60-min incubation, 100-µL aliquots were collected at 10-min intervals and centrifuged at 13,800× *g* for 3 min. The titer of unadsorbed phages in the supernatant was determined using the double-agar overlay method. These values were used to calculate the percentage of unadsorbed phages and construct adsorption curves [28]. Experiments were performed using three independent biological replicates.

### 4.5. One-Step Growth Curve

One-step growth assay was performed as described previously [27]. Mid-logarithmic-phase cells were infected at an MOI of 0.01 in a total volume of 0.9 mL. After 40 min, unadsorbed phage particles were inactivated by adding 0.1 mL of 100 mM ferrous ammonium sulfate (Sigma-Aldrich, St. Louis, MO, USA), followed by incubation for 5 min at room temperature. Cells were then centrifuged at 10,000× *g* for 3 min, and the pellet was resuspended in 1 mL Middlebrook 7H9 broth.

The resulting suspension was used to quantify infected cells and generate a one-step growth curve. The number of infected cells was determined using the double-agar overlay method. To generate the growth curve, 100 µL of the suspension was diluted 100-fold in fresh Middlebrook 7H9 broth. Aliquots were collected at 30-min intervals for 3 h and centrifuged at 13,800× *g* for 3 min, after which the titer of free phage in each supernatant was determined using the double-agar overlay method. Burst size was calculated by dividing the total number of released phage particles by the number of infected bacterial cells. The experiment was performed in triplicate.

### 4.6. Host-Range Determination

Phage host ranges were determined by spot testing using the double-agar overlay method [27]. Test cultures were washed twice with Middlebrook 7H9 broth to remove Tween 80. Tenfold serial dilutions of each phage were prepared in MP buffer from stocks with initial titers of 10^7^–10^10^ PFU/mL and spotted onto bacterial lawns in 2-µL volumes. The same dilution series was used for all tested hosts. Plates were incubated for 48 h for rapidly growing mycobacteria and for up to 3 weeks for slowly growing mycobacteria.

### 4.7. Assessment of Lysogen Formation

Lysogeny frequency was assessed using a standard protocol [https://phagesdb.org/workflow/FurtherDiscovery/ (accessed on 19 March 2013)]. Aliquots containing 100 µL of each bacteriophage at 10^10^ PFU/mL were distributed uniformly over the surface of plates containing 2% agar. Tenfold serial dilutions (10^−3^–10^−7^) were prepared from an overnight culture of *M. smegmatis* mc(2)155. A 100-µL volume of each dilution was added to molten soft agar, and the mixture was poured onto plates with or without phage. After incubation at 37 °C for 6 days, colonies were counted. Lysogeny frequency was calculated as follows: lysogeny (%) = (CFU on phage-containing plates/CFU on control plates) × 100.

Ten individual *M. smegmatis* mc(2)155 colonies recovered from the lysogeny-frequency assays for each of Shep and Cat were subcultured twice to remove free phage particles. The presence of a prophage in the bacterial genome was assessed by PCR using primers flanking the predicted integration site (Figure S2C). Phage DNA was also tested with primers specific to a region of the Cat genome. One colony recovered from the Cat lysogeny-frequency assay was subjected to whole-genome sequencing. The same strain was passaged a further ten times in the absence of phage to minimize potential effects of transient overexpression of genes associated with phage resistance.

Immunity to superinfection was assessed using mycobacteriophages Cat and Shep, as well as Ground-D (subcluster P1) and YPΔ47 (subcluster K4, Ref. [30]). Phage titers were determined as described above.

### 4.8. Phage Stability Under Different Conditions

To assess pH stability, 100 µL of phage lysate at 7 × 10^9^ PFU/mL was mixed with 900 µL of saline adjusted to pH values ranging from 2 to 12. Samples were incubated at 20 °C for 24 h. Thermal stability was assessed by incubating lysates at −20, 4, 20, 37, 45 or 55 °C for 24 h.

Sensitivity to ultraviolet irradiation was assessed by placing 700 µL of phage lysate of known titer in a quartz cuvette positioned 5 cm from a 254-nm, 15-W germicidal lamp. Samples of 10 µL were collected at 20-min intervals over 180 min. In all stability experiments, phage titers were determined by spot assay on *M. smegmatis* mc(2)155 after treatment. Experiments were performed in triplicate.

### 4.9. DNA Extraction and Whole-Genome Sequencing

Phage genomic DNA was isolated by phenol–chloroform extraction, while bacterial genomic DNA was extracted using the KingFisher Flex system (Thermo Fisher Scientific, Waltham, MA, USA) with a magnetic bead-based protocol. DNA concentrations were measured using the Quant-iT High-Sensitivity DNA Assay Kit (Thermo Fisher Scientific, Waltham, MA, USA). Sequencing libraries were prepared from 100 ng of purified DNA using the KAPA HyperPlus Kit (Roche, Rotkreuz, Switzerland) according to the manufacturer’s instructions. Library products were purified using KAPA HyperPure Beads (Roche, Rotkreuz, Switzerland). Library quality and fragment-size distributions were assessed using an Agilent 2100 Bioanalyzer with a High-Sensitivity DNA Chip (Agilent Technologies, Santa Clara, CA, USA). Libraries were quantified using the Quant-iT High-Sensitivity DNA Assay Kit (Thermo Fisher Scientific, USA). Sequencing was performed on a NextSeq 1000 platform (Illumina, Waltham, MA, USA) using a NextSeq 1000/2000 P2 Reagents Kit (200 cycles), v.3, with 2% PhiX (Illumina, Waltham, MA, USA) added as an internal control. The raw sequencing reads for the wild-type *M. smegmatis* mc(2)155 strain and its phage-resistant mutant are available in the NCBI Sequence Read Archive (SRA) under BioProject accession number PRJNA1499085.

### 4.10. Bioinformatic Analyses

For phage genome assembly, short reads were quality-filtered and trimmed using fastp v.0.23.4 [31] with the parameters -q 20 -5 -3 -l 100 -t 2 --detect_adapter_for_pe --dedup. The resulting reads were subsampled to 50× coverage using rasusa v.2.0.0 [32], assuming a genome size of 0.065 Mb. Genomes were assembled using SPAdes v.4.0.0 in metaviral mode (--metaviral; Ref. [33]). Assembly quality and completeness were assessed using CheckV v.1.0.1 [34]. To confirm read support and determine final sequencing coverage, trimmed reads were mapped back to the assemblies using BWA-MEM2 v.2.2.1 [35], and the resulting alignments were processed using SAMtools v.1.17. Open reading frames were predicted and proteins were functionally annotated using Pharokka v.1.7.5 [36].

Phylogenetic relationships were inferred using VirusDicer v.0.1.1 (https://github.com/dbespiatykh/VirusDicer (accessed on 1 August 2026)) with 1000 bootstrap replicates, based on 779 unique genomes from the Actinobacteriophage Database (accessed 1 April 2026). The resulting tree was visualized in R v.4.6.1 (Ref. [37], https://www.R-project.org/) using ape v.5.8.1 [38], cowplot v.1.2.0 [39], ggnewscale v.0.5.2 [40], ggplot2 v.4.0.3 [41], ggstar v.1.0.6 [42], ggtree v.4.2.0 [43], ggtreeExtra v.1.22.1 [44] and phytools v.2.5.2 [45].

To characterize repressor diversity, homologs were searched in 117 subcluster A3 genomes using BLASTn v.2.15.0+ [46] with one representative sequence from each provisional variant as a query. Full-length or near-full-length sequences (n = 101) were aligned using MAFFT v.7.520 [47,48], and pairwise nucleotide identities were calculated. A maximum-likelihood tree was inferred with IQ-TREE v.2.3.5 [49] under the HKY + F+ G4 model with 1000 ultrafast bootstrap and 1000 SH-aLRT replicates.

Phage synteny was assessed using pairwise BLASTp v.2.15.0+ comparisons [46] and visualized in R using gggenomes v.1.1.3 [50].

To identify the phage integration site, nucleotide BLASTn searches [46] were performed against the genome of *M. smegmatis* mc(2)155 (CP000480) using the assembled phage genomes as queries.

To identify mutations associated with phage resistance, reads were quality-filtered and adapter-trimmed using fastp v.1.1.0 [51], and read quality was assessed with FastQC v.0.12.1 (https://www.bioinformatics.babraham.ac.uk/projects/fastqc/ (accessed on 1 April 2026)) and MultiQC v.1.33 [52]. Trimmed reads were mapped to the *M. smegmatis* mc(2)155 genome (CP000480) using BWA-MEM v.0.7.19 [53]; alignments were sorted and indexed with SAMtools v.1.22.1 [54], and duplicates were marked with Picard MarkDuplicates v.3.4.0 (https://broadinstitute.github.io/picard/ (accessed on 1 April 2026)) and excluded from variant calling. Variants were called with BCFtools v.1.23.1 [54] under a haploid model, normalized to biallelic records, and filtered to retain sites with QUAL ≥ 20 and read depth ≥ 10. A joint call of all sequenced clones was additionally performed to compare them at identical genomic positions in a single multi-sample VCF file. The analysis was implemented as a Nextflow v.26.04.3 [55] workflow (asm-lite v.2.0.0, https://github.com/KiselevSI/asm-lite, (accessed on 1 April 2026)).

## 5. Conclusions

The phages described here provide a valuable resource for investigating evolutionary processes within subcluster A3 and broaden our understanding of mycobacteriophage genome plasticity. Their ability to infect *Mtb* at efficiencies approaching those of the most active members of this subcluster makes them promising candidates for therapeutic development. Importantly, the identified deletions are naturally occurring, a relevant consideration under regulatory frameworks governing phage preparations intended for therapeutic use. Phage Cat, which showed the greatest lytic activity, warrants further investigation as a potential component of therapeutic phage cocktails, including evaluation of its efficacy in vivo and identification of strategies to improve its yield.

## Figures and Tables

**Figure 1:**
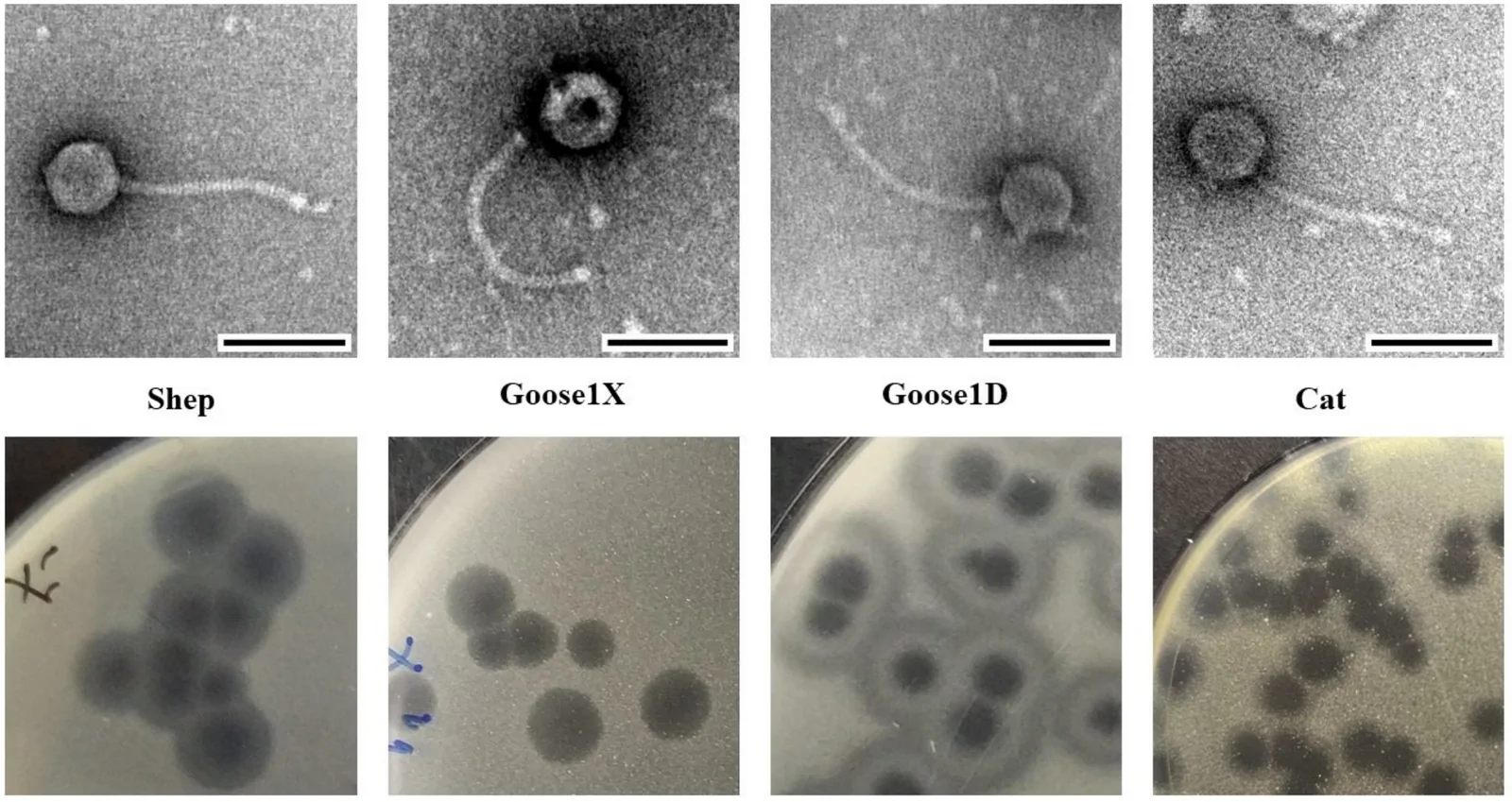
Morphological characteristics of four newly isolated mycobacteriophages. Top, transmission electron micrographs; scale bar, 100 nm. Bottom, plaques formed on *M. smegmatis* mc(2)155 after 24 h of incubation.

**Figure 2:**
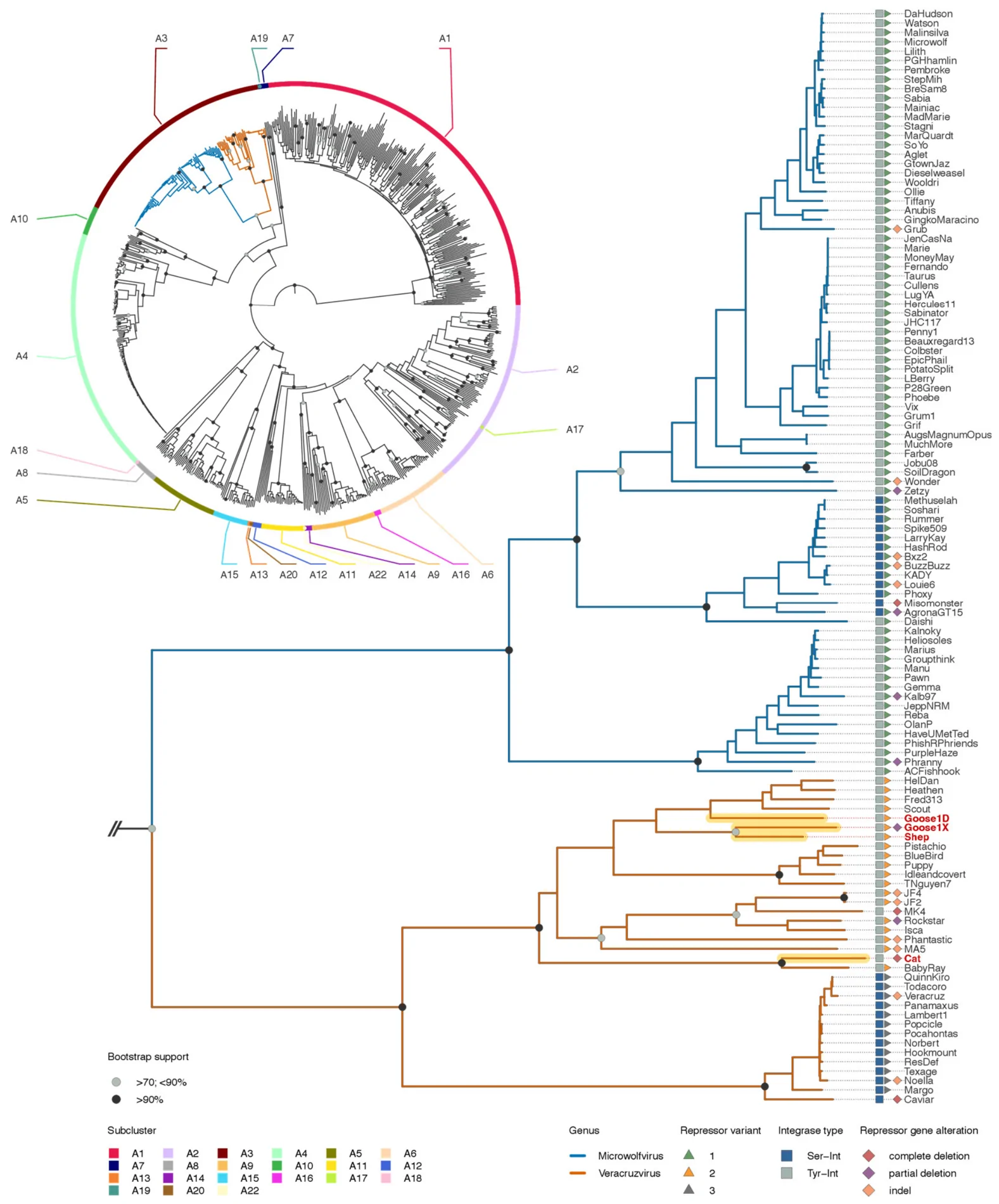
Phylogenetic tree of cluster A mycobacteriophages. A midpoint-rooted BioNJ tree was inferred from proteome-wide Dice distances among 779 cluster A mycobacteriophage genomes using 1000 bootstrap replicates. Bootstrap support at internal nodes is indicated by grey (70–<90%) or black (≥90%) circles. Colors in the circular overview denote the different subclusters within cluster A. The accompanying rectangular subtree provides an enlarged view of subcluster A3. Colored branches indicate members of the genera *Microwolfvirus* and *Veracruzvirus*. Squares show integrase type (serine or tyrosine integrase), triangles indicate repressor variants 1–3, and diamonds denote repressor-gene alterations classified as complete deletion, partial deletion or indel. Cat, Goose1D, Shep and Goose1X are emphasized by bold red labels and highlighted branches.

**Figure 3:**
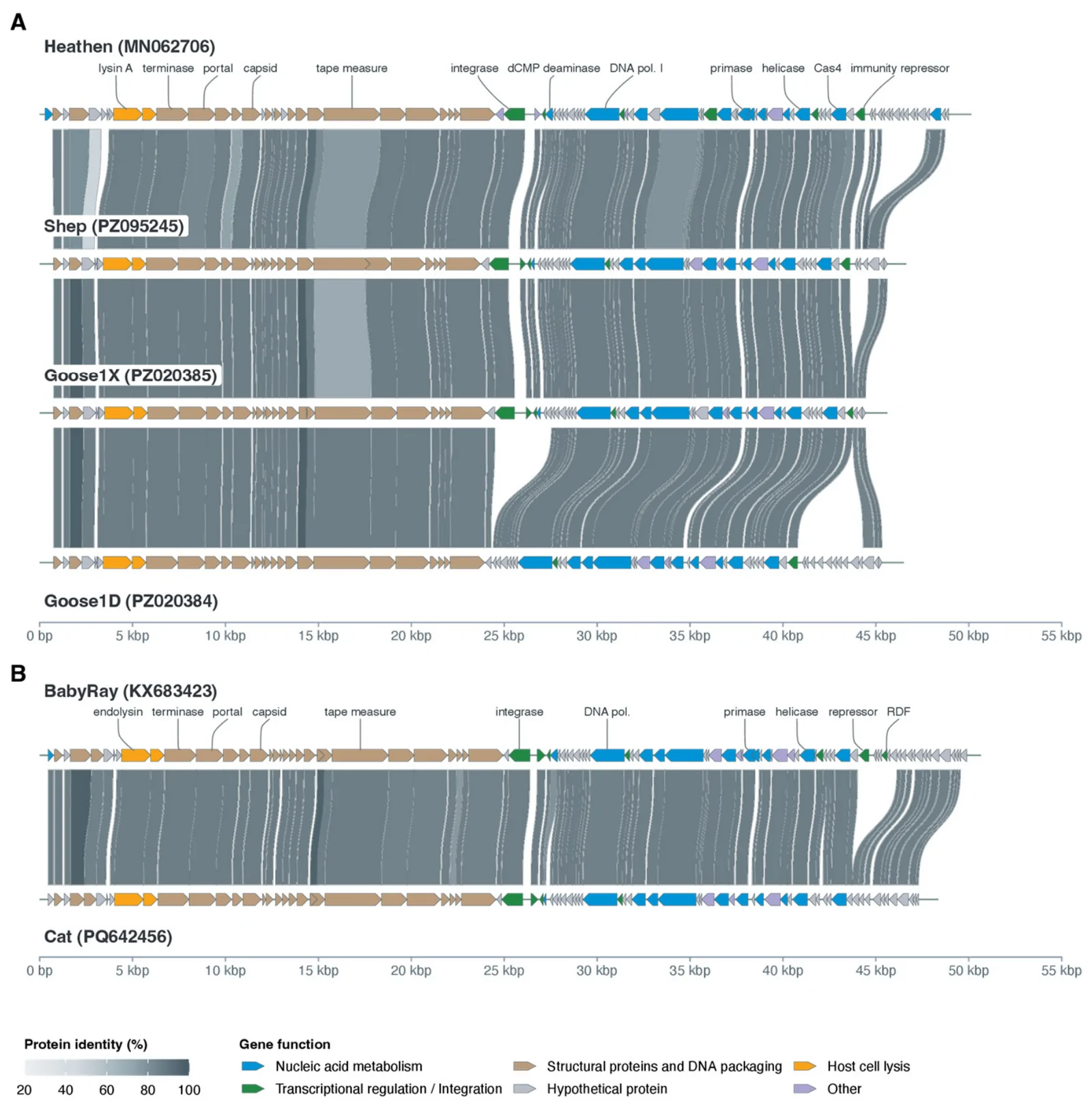
Comparative genome organization and protein-level synteny among selected A3 mycobacteriophages. (**A**) Four-genome comparison of Heathen, Goose1D, Goose1X, and Shep. (**B**) Pairwise comparison of BabyRay and Cat. Gene arrows indicate annotated-feature position and orientation and are colored by functional category. Curved ribbons connect best-hit BLASTp v.2.15.0+ homologues (E-value ≤ 1 × 10^−5^); ribbon shading represents amino-acid identity according to the scale.

**Figure 4:**
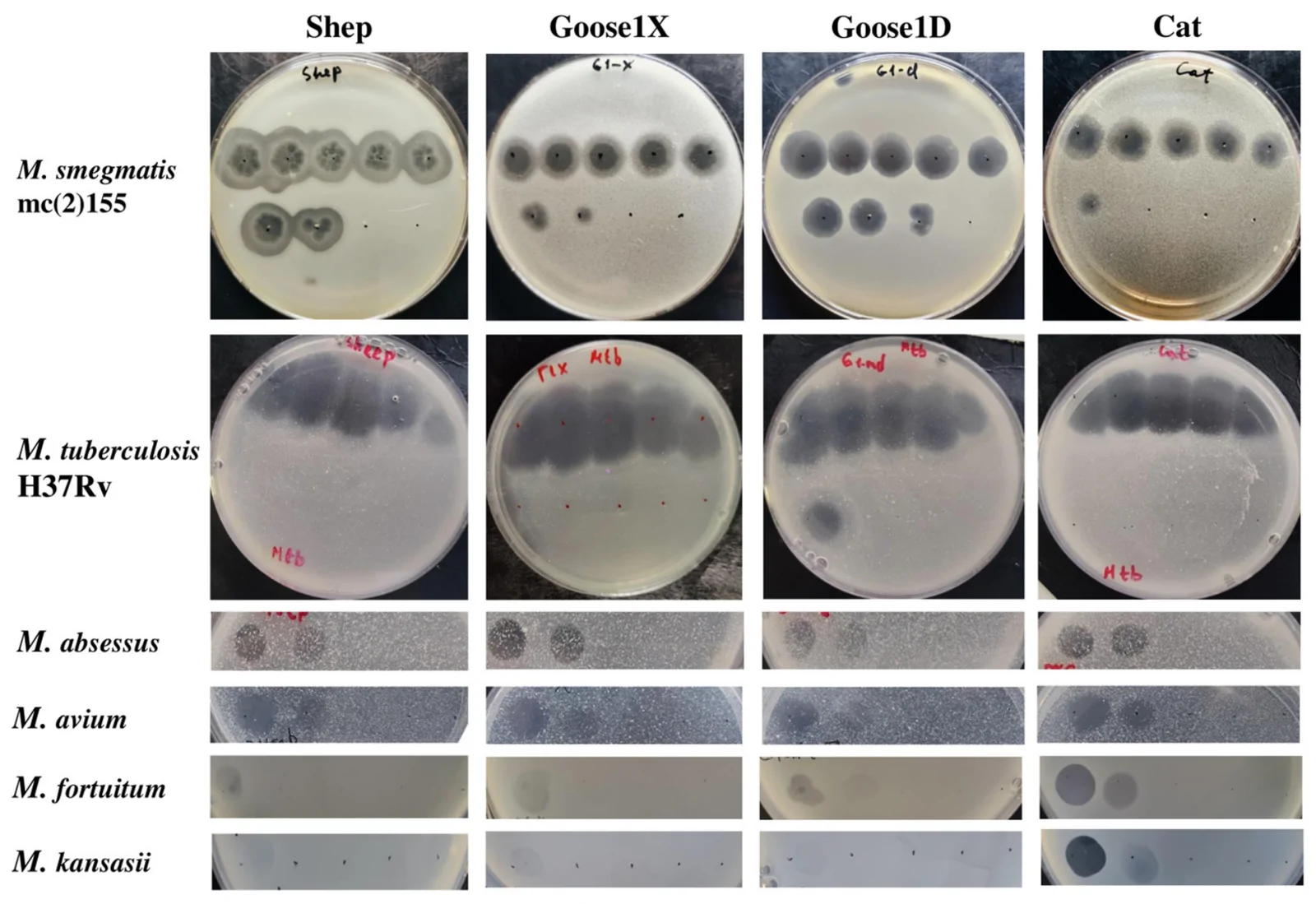
Host ranges of phages Shep, Goose1X, Goose1D, and Cat across different mycobacterial strains. Tenfold serial dilutions of each phage stock (initial titers 10^7^–10^10^ PFU/mL) were prepared; 2 µL of each dilution was spotted onto bacterial lawns. The first spot in each row corresponds to the undiluted stock, followed by the dilution series. The same dilution series was used for all tested hosts within a given phage experiment.

**Figure 5:**
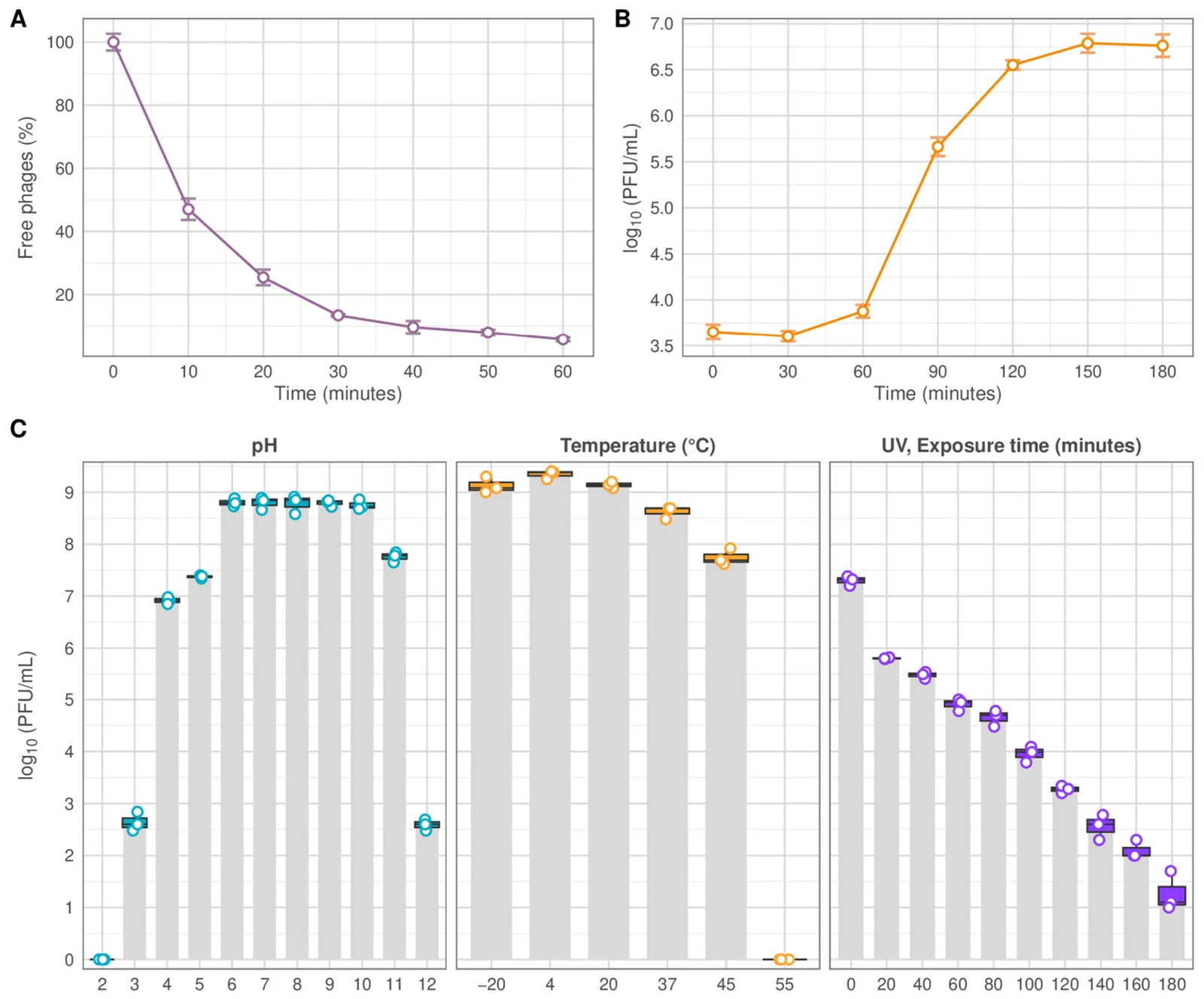
Life-cycle characteristics and physicochemical stability of phage Cat. (**A**) Adsorption curve. (**B**) One-step growth curve. (**C**) Effects of pH, temperature and ultraviolet exposure on phage infectivity.

**Figure 6:**
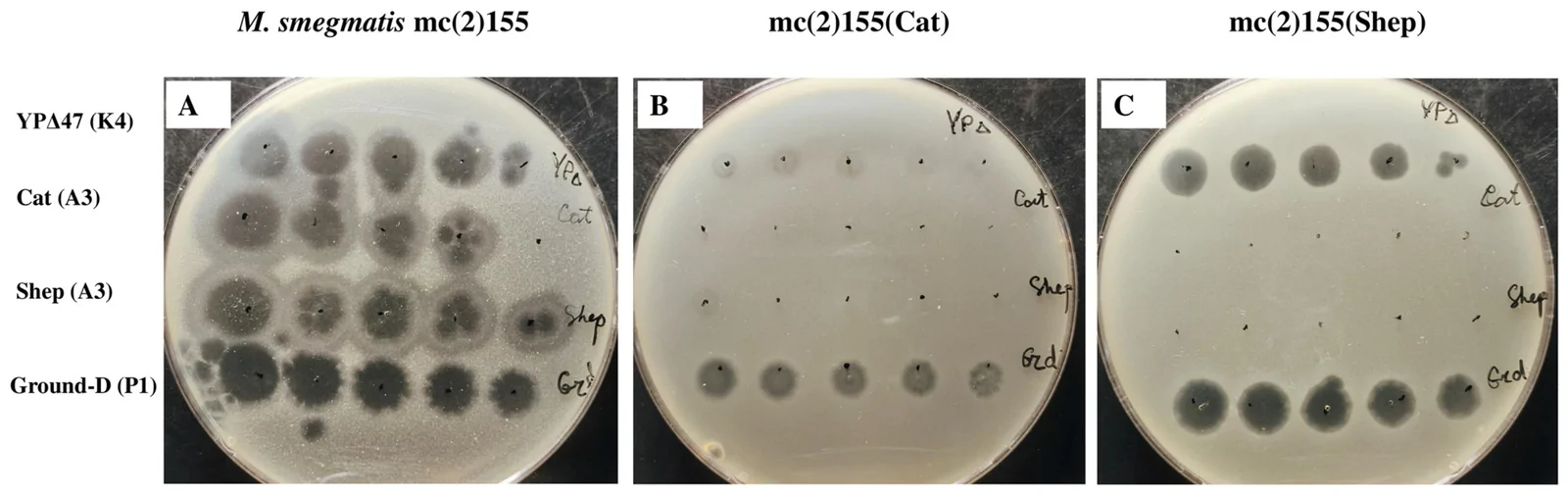
Superinfection immunity profile of a mutant *M. smegmatis* strain recovered after Cat infection and a stable mc(2)155(Shep) lysogen. (**A**) Spot assay on a lawn of *M. smegmatis* mc(2)155 (control). (**B**) Spot assay on the mutant strain recovered after Cat infection. (**C**) Spot assay on the stable mc(2)155(Shep) lysogen. Tested phages and their cluster/subcluster affiliations are indicated on the left.

**Table 1:** Genomic characteristics of subcluster A3 phages.

Phage	Length (bp)	G + C (%)	ORFs	*tRNA*	Accession No
Shep	46,797	63.8	74	2	PZ095245
Goose1X	45,626	63.9	71	2	PZ020385
Goose1D	46,510	63.8	76	2	PZ020384
Cat	48,385	63.6	77	1	PQ642456

## Data Availability

The original genome sequences of the newly isolated phages presented in this study are openly available in the NCBI GenBank database under accession numbers PZ095245, PZ020385, PZ020384, and PQ642456. Additionally, the whole-genome sequencing reads of the phage-resistant *Mycobacterium smegmatis* mutants generated and analyzed in this study are available in the NCBI Sequence Read Archive (SRA) under BioProject accession number PRJNA1499085.

## Supplementary Materials

The following supporting information can be downloaded at: https://www.mdpi.com/article/10.3390/ijms27198476/s1. References [56,57] are cited in the Supplementary Material.

## Abbreviations

The following abbreviations are used in this manuscript:

*Mtb*	*Mycobacterium tuberculosis*
NTM	Nontuberculous mycobacteria
EOP	Efficiency of plating
ORF	Open reading frames
CFU	Colony-forming unit
PFU	Plaque-forming unit
MOI	Multiplicity of Infection
