# Supplementary material for: Characterization of Novel Mycobacteriophages of Subcluster A3: Evolutionary Plasticity of the Lysogeny Module and Lytic Potential
Source: Int J Mol Sci. 2026 Sep 23;27(19):8476. doi: 10.3390/ijms27198476 (PMC13664763; doi:10.3390/ijms27198476)
Supplement: Supplementary file 1 [file ijms-27-08476-s001.zip › Supplementary Materials.pdf]

Supplementary Materials: Figure S1: Activity of mycobacteriophage Cat against clinical Mtb isolates. Spot assays were performed using tenfold serial dilutions of phage lysates with an initial titer of approximately 10^9 PFU/mL. Phylogenetic lineages were assigned according to Shitikov and Bespiatykh (2023; [56]). Drug-resistance profiles were inferred using TB-Profiler (Phelan et al., 2019; [57]). Antibiotic abbreviations are as follows: RIF, rifampicin; INH, isoniazid; EMB, ethambutol; PZA, pyrazinamide; MFX, moxifloxacin; LVX, levofloxacin; BDQ, bedaquiline; DLM, delamanid; PMD, pretomanid; LZD, linezolid; STR, streptomycin; AMK, amikacin; KAN, kanamycin; CAP, capreomycin; and ETH, ethionamide; Figure S2. PCR-based analysis of prophage integration in mc(2)155(Shep) and putative mc(2)155(Cat) strains. (A) Primer localization scheme across the parental strain, the mc(2)155(Shep) lysogen, the putative mc(2)155(Cat) strain, and the phage Cat genome. The integration site (MSMEG_4676, tRNA-Gly / attB) is highlighted in orange. Numbered primer pairs (1–6) are shown with their expected amplicon sizes. (B) Agarose gel electrophoresis of PCR products. I–IV correspond to the templates and 1–6 to the primer pairs, as defined in panel A. M, 100 bp DNA {ladder (100–1000 bp; the brightest band is 500 bp). (C) Sequences of the primers used. Figure S3. Repressor sequence diversity among subcluster A3 phages. A, Maximum-likelihood phylogeny of 101 high-confidence full-length or near-full-length repressor coding sequences. Tip labels and points are colored according to repressor variant (type 1, green; type 2, orange; type 3, gray), and black internal circles indicate ultrafast bootstrap support >=95%. Branch lengths represent substitutions per site. B, Global pairwise nucleotide identities among the three representative repressor sequences supplied for the provisional variants. C, Distributions of pairwise nucleotide identity within and between variants across the 101 high-confidence sequences; Figure S4. Adsorption of phage Cat to the *M. smegmatis* phage-resistant mutant. Adsorption curves were generated at an MOI of 0.01. Data are presented as mean ± SD from three independent biological replicates.
